# Pilot study of dovitinib in patients with von Hippel-Lindau disease

**DOI:** 10.18632/oncotarget.25171

**Published:** 2018-05-04

**Authors:** Patrick Pilié, Elshad Hasanov, Surena F. Matin, Ashley H. Henriksen Woodson, Valerie D. Marcott, Shelly Bird, Rebecca S. Slack, Gregory N. Fuller, Ian E. McCutcheon, Eric Jonasch

**Affiliations:** ^1^ Department of Genitourinary Medical Oncology, Division of Cancer Medicine, University of Texas MD Anderson Cancer Center, Houston, TX 77030, USA; ^2^ Department of Internal Medicine, McGovern Medical School, The University of Texas Health Science Center at Houston, Houston, TX 77030, USA; ^3^ Department of Urology, Division of Surgery, The University of Texas MD Anderson Cancer Center, Houston, TX 77030, USA; ^4^ Department of Clinical Cancer Genetics, University of Texas MD Anderson Cancer Center, Houston, TX 77030, USA; ^5^ Department of Biostatistics, The University of Texas MD Anderson Cancer Center, Houston, TX 77030, USA; ^6^ Department of Pathology, The University of Texas MD Anderson Cancer Center, Houston, TX 77030, USA; ^7^ Department of Neurosurgery, Division of Surgery, The University of Texas MD Anderson Cancer Center, Houston, TX 77030, USA

**Keywords:** von Hippel-Lindau, hemangioblastomas, tyrosine kinase inhibitor, dovitinib, fibroblast growth factor receptor

## Abstract

Von Hippel-Lindau (VHL) disease is an autosomal dominant disease occurring in 1 in 35,000 births and leads to an increased risk of a phenotypically diverse array of tumor types including, but not limited to, clear cell renal cell carcinoma (ccRCC) and hemangioblastomas (HBs). Previous studies of patients with VHL disease treated with the tyrosine kinase inhibitor (TKI) sunitinib did not show clinical response in HBs. Interestingly, VHL-related HBs displayed increased fibroblast growth factor receptor 3 (FGFR3) protein expression when compared to VHL-related ccRCCs. Therefore, in this pilot study, we assessed the safety and efficacy profile of TKI 258 (dovitinib), a multi-tyrosine kinase inhibitor of VEGF receptor and fibroblast growth factor (FGF), in patients with VHL disease who had measureable HBs. The trial was stopped after six patients enrolled after the toxicity stopping rule was triggered. With regards to safety, 6/6 subjects had at least one adverse event (AE). Best response in 6/6 subjects was stable disease (SD) in HBs. While the negative safety and efficacy results of this pilot study do not favor the use of dovitinib for the treatment of asymptomatic HBs in VHL disease patients, further investigation into alternative scheduling and other FGFR inhibitors for the treatment of HBs in VHL disease patients is warranted given the promising pre-clinical and molecular data.

## INTRODUCTION

Germline mutations in the von Hippel-Lindau (VHL) gene, a tumor suppressor found on chromosome 3p25, are inherited in an autosomal dominant fashion giving way to the development of a spectrum of tumor types including: renal and pancreatic cysts, clear cell renal cell carcinoma (ccRCC), hemangioblastomas (HB), pheochromocytomas (PCC), retinal HBs, and pancreatic neuroendocrine tumors (pNET) [1–3]. VHL disease occurs in approximately 1 in 35,000 births, and the morbidity and mortality associated with VHL disease primarily centers around the neurologic complications of HBs and the progression of ccRCC, with HBs being the most frequently seen lesion in VHL-disease, occurring in over 70% of patients [1, 4]. A majority of VHL-related HBs will grow over the patient's life and left untreated lead to neurologic symptoms due to mass effect [5, 6]. The current primary treatment for symptomatic HBs or threatening HBs is surgical resection. We lack systemic treatment options for non-RCC VHL-related tumor types, including HBs.

VHL lesions, despite different anatomic origins, are in general highly vascular owing to the loss of the underlying anti-angiogenic function of the *VHL* gene. The protein product of *VHL* gene, pVHL, in normoxic conditions recognizes the oxygen-dependent prolyl-hydroxylation of hypoxia inducible factor α (HIF) and targets HIF for ubiquitylation and subsequent proteasomal degradation [4, 7]. However, in hypoxic conditions or in cancer cells lacking pVHL due to mutational loss, HIFα dimerizes with HIFβ. This HIF heterodimer then transactivates pro-angiogenic hypoxia-response elements including key proteins in cell growth and energy metabolism such as vascular endothelial growth factor (VEGF), platelet derived growth factor (PDGF), fibroblast growth factor (FGF), and glucose transporter 1&3 (GLUT1&3) [4, 8]. Given that VHL inactivation leads to inappropriate angiogenesis in both sporadic and germline VHL-disease associated lesions, tyrosine kinase inhibitors against the VEGF pathway, such as sunitinib and pazopanib, are approved treatment approaches for metastatic ccRCC and are just some of the inhibitors being actively investigated for treatment of VHL disease.

A pilot study of sunitinib in 15 patients with germline *VHL* mutations with measurable VHL-associated lesions showed that the drug had manageable toxicity and that 33% (6/18) of RCC lesions showed partial response; however, 0/21 HB lesions showed response [9]. The reason why organ specific VHL-related lesions respond differently to anti-angiogenic therapy is unclear, though RCC and HBs are inherently different as HBs do not represent true cancer and lack metastatic potential. Preclinical studies in mouse models of late stage pancreatic islet cell tumors have shown tumor resistance to VEGF via hypoxia-mediated induction of proangiogenic factors other than VEGF, including members of the FGF family [10]. In this same study, protein expression analyses of select proangiogenic pathways via laser-scanning cytometry was performed on 20 VHL-related HBs not treated on the study and compared to 20 RCC tumors. Interestingly, the RCC tissues displayed higher expression levels of pVEGFR-2 when compared to HBs; however, protein expression levels of phosphorylated fibroblast growth factor receptor substrate-2 (FGFR2) and FGFR3 were higher in HBs compared to RCCs [9]. Dovitinib (TKI 258, Novartis) is a multi-TKI that inhibits FGFR, VEGFR, and PDGFR. A phase II study of dovitinib 500 mg/day (5 days on/2 days off dosing) in 67 metastatic RCC patients, most of which had received prior VEGFR TKI and/or mTOR inhibitor, showed this regimen was tolerable and displayed disease control rate of 56.4% with median progression-free survival (PFS) and overall survival (OS) at 3.7 and 11.8 months, respectively [11]. In this study, dovitinib induced inhibition of VEGFR and FGFR in patient tissue samples. The differences seen in prior studies in endothelial angiogenic receptor expression levels in HBs combined with the biologic targets of dovitinib prompted the current pilot phase II study to assess the safety and efficacy of dovitinib in individuals with VHL disease and measureable HBs.

## RESULTS

### Patients

From November 2012 to October 2013, patients with clinically or genomically defined VHL disease and a measureable HB were recruited to participate in the trial. 83% of the patients had cerebellar HBs, 66% brainstem HBs and 50% retinal HBs. Patients’ demographics and clinical manifestations are summarized in Table 1. 2/6 of the patients had received prior systemic therapy with tyrosine kinase inhibitors with both patients having been treated with sunitinib and pazopanib prior to enrolling on this trial. The study was stopped after six patients were enrolled due to activation of the toxicity stopping rule.

**Table 1:** Patient demographics and clinical characteristics

Characteristics	Patients (*N* = 6)
Age, years; median (range)	44 (18–61)
Sex, *n* (%)	
Male	5 (83)
Female	1 (17)
Race, *n* (%)	
African American	1 (17)
Caucasian	3 (50)
Asian	0
Hispanic	2 (33)
Other	0
VHL disease manifestation, *n* (%)	
Cerebellar hemangioblastoma	5 (83)
Brainstem hemangioblastoma	4 (66)
Retinal hemangioblastoma	3 (50)
Renal cell carcinoma	2 (33)
Pancreatic cysts	2 (33)
Other	0
Prior systemic TKI therapy, *n* (%)	2 (33)

### Dovitinib treatment

All six patients completed at least two cycles of therapy. The average time on therapy was 5.3 months (range 2–11months). One patient discontinued the treatment due to noncompliance. Another patient discontinued due to progression. 50% of the patients discontinued due to side effects including vomiting, rash, and dyspnea. Despite dose reduction, most patients could not tolerate the treatment. One patient completed the full six cycles at full dose but chose not to continue therapy due to rash side effect. Table 2 describes the dovitinib treatment for each patient.

**Table 2:** Efficacy of dovitinib treatment for hemangioblastomas in Von Hippel Lindau patients

Patient	Best response	Discontinuation reason	Cycles completed
A	SD	Non-compliant/AE	12
B	SD	AE	2
C	SD	Progression/AE	6
D	SD	AE	4
E	SD	AE	2
F	SD	Completed study	8

Abbreviations: SD = stable disease; AE = adverse event.

### Toxicity

With regards to safety, all patients had at least one adverse event (AE) with the most common AEs being rash, diarrhea, and fatigue. Table 3 describes the most common adverse events. Maculopapular rash was the most common and severe AE. One patient had grade 3 rash and this was the only grade 3 AE in the study. There were no other serious adverse events reported and no deaths during the investigation.

**Table 3:** Frequency and grade of adverse events in dovitinib-treated patients

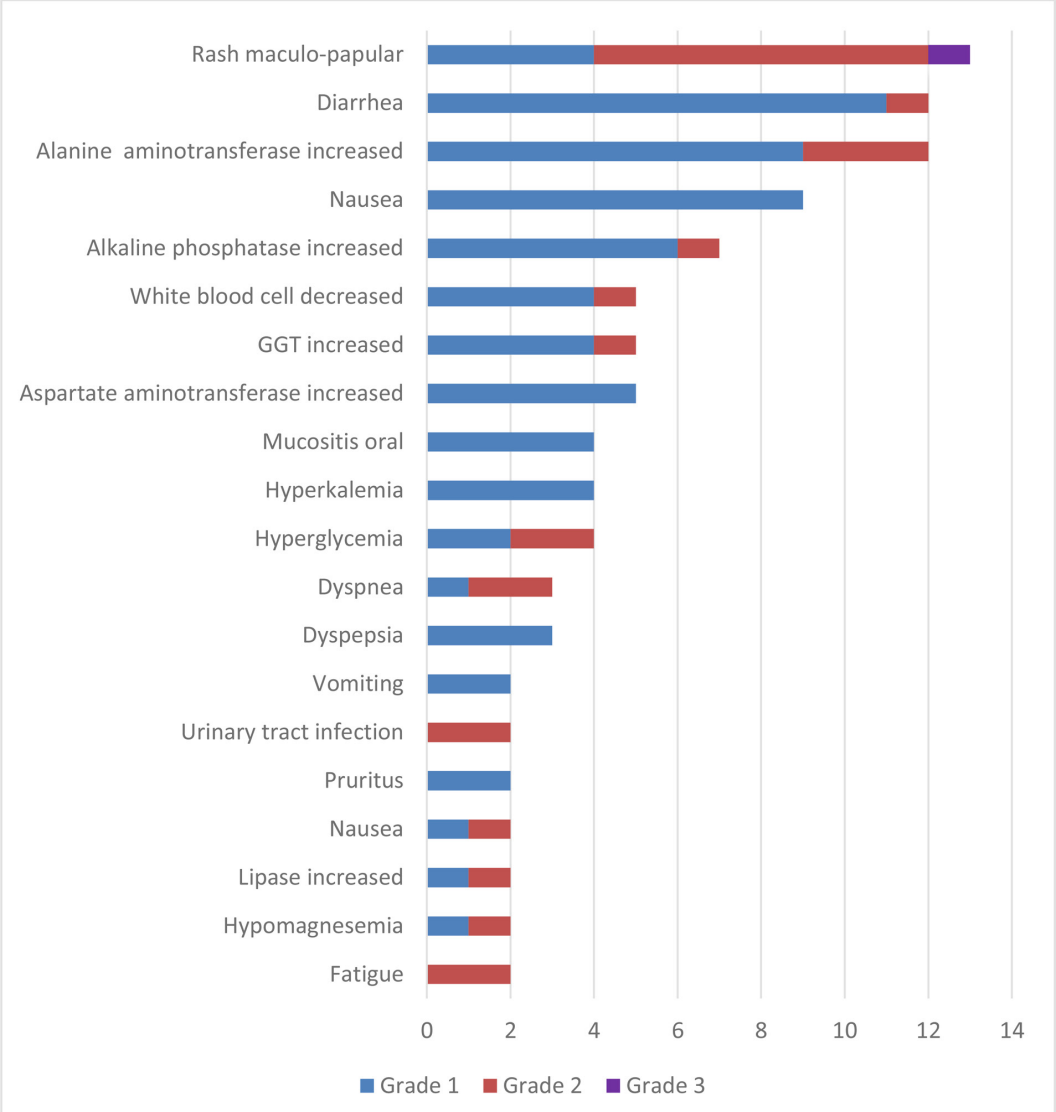

### Efficacy

Of the six patients treated on the study, no patient demonstrated a RECIST response. All patients displayed best response of stable disease (SD) in HBs. One of the patients eventually had progression while on therapy.

## DISCUSSION

This is the first study to evaluate dovitinib therapy to treat VHL-related HBs, with a primary endpoint of safety and a secondary endpoint of efficacy. This pilot study did not show a favorable safety or efficacy profile in VHL-disease patients with measureable HBs, despite the strong biologic rationale based on prior molecular data showing overexpression of FGFR protein in HBs compared to RCC tumors from VHL disease patients. The most frequent adverse events in our study were similar to a prior study of dovitinib in patients with metastatic RCC with at least one AE occurring in 6/6 of the patients on our study and 3/6 patients stopping therapy due to AEs [11]. The lack of response in HBs in this patient population treated with dovitinib is surprising, and molecular profiling of HB tissue would be extremely useful to help understand the biologic underpinnings for this lack of efficacy. However, given that the patients in this study had measurable, non-threatening HBs, pre-treatment tissue acquisition for molecular biomarker profiling was not possible. Future prospective comprehensive profiling of VHL-related and sporadic HBs is warranted to guide treatment and prevention strategies. VHL-related HBs remain a leading cause of morbidity and mortality in VHL disease patients. Surgical resection is currently the mainstay of managing these HBs if symptomatic or symptoms are impending. Systemic therapies such as sunitinib, a VEGF TKI, that are effective in VHL-associated RCC have not shown benefit for HBs [9, 12]. Prospective biomarker studies of differentially expressed protein(s) in HBs versus RCCs versus other non-RCC lesions can greatly inform future trial design and treatment selection/sequence. It is still possible that targeting FGFR signaling could have an impact on HB progression and symptoms; however, our study is limited by small sample size, the inability to obtain paired molecular data, and possible lack of FGFR specificity of dovitinib. A Phase II trial of pazopanib in patients with VHL disease with measurable lesions (NCT01436227) has shown early promising results with responses in RCCs, pancreatic lesions, and HBs with an acceptable safety profile [13]. Comprehensive genomic, transcriptomic, and proteomic evaluation of HBs that respond on this study will inform future biomarker design and selective treatment strategies.

## METHODS

### Study design

This is a single center, single-arm, open-label, phase II study was approved by University of Texas MD Anderson Cancer Center ethics committee. The study was registered with ClinicalTrials.gov (NCT01266070). Written consent was obtained from all patients prior to joining the study.

### Patient population

Individuals with VHL disease who had measureable HBs with no immediate risk of needing intervention were recruited to this study. All patients were age 18 years or older with genetically confirmed VHL disease or a clinical diagnosis of VHL diseaes with at least one measureable HB (>0.5 cm) in the brain or spine which did not have an immediate need for intervention. Patients who had undergone prior therapy for VHL lesions were included as long as other criteria were met. Additional criteria included European Co-operative Oncology Group Performance Status (ECOG) performance status 2 or better, and normal organ and marrow function. Patients who had chemotherapy or radiation within 4 weeks prior to study drug start time or those patients who had not recovered from adverse events from prior agents were excluded. In addition, patients with metastatic disease of any kind, hypertension uncontrolled by medication, cardiac dysrhythmias of grade 2 or higher, grade 3 hemorrhage within 4 weeks of starting study drug, pregnant women, and known HIV-positive patients on antiretrovirals were excluded. Additional exclusive criteria were any of the following within the 6 months prior to study drug administration: myocardial infarction, severe/unstable angina, coronary/peripheral artery bypass graft, symptomatic congestive heart failure, cerebrovascular accident or transient ischemic attack, or pulmonary embolism.

### Treatment

Dovitinib 500 mg/day was given in a 4-week cycle on a 5 day on/2 day off schedule. In the absence of adverse events (AE), treatment lasted 6 cycles or until disease progression, unacceptable adverse event(s), withdrawal from the study, or general or specific changes in the patient's condition rendered the patient ineligible to receive further treatment. For patients who were unable to tolerate the protocol-specified dosing, dose interruption and modifications were permitted. Treatment was held for any drug-related >Grade 2 toxicity events until the severity is reduced to Grade 1 or less and was then resumed with a dose reduction. Based on the worst toxicity demonstrated in the last dose, dovitinib dosage was reduced to 400 mg/day and then 300 mg/day. Dose reduction below 300 mg was not allowed. Patients with toxicities that failed to resolve to <Grade 1 within 21 days were taken off study.

### Clinical evaluations

Evaluation of response to treatment using RECIST was done every 8 weeks including imaging, laboratory, and clinical evaluation. Baseline and follow-up evaluations of target lesions were carried out by using computed tomography (CT) scanning or magnetic resonance imaging (MRI), as appropriate. Spiral CT scanning, involving multiphase contrast-enhanced studies of the abdomen and pelvis with thin cuts (≤5 mm) of the adrenals, kidneys, and pancreas, was predominantly used to follow pancreatic and renal lesions. Dynamic contrast-enhanced and diffusion-weighted MRI sequences of the brain and spinal column were used for evaluating CNS HBs. We used RECIST but modified them to uncouple target organ systems, and each organ system was evaluated separately. Summation of size was carried out separately for lesions in the kidney and CNS (brain + spine), and retina, and these measurements were used to compare changes from baseline size. Toxicity and adverse events were recorded using Common Terminology Criteria for Adverse Events (CTCAE) Version 4.0.

### Endpoints

The primary objective was to evaluate safety of treatment with dovitinib for 6 months in patients with VHL who had a measurable HB undergoing surveillance. The secondary objective was to investigate the utility of FGFR, VEGFR and PDGFR pathway blockade in VHL patients with HBs and other VHL related lesions and to assess response rate of HBs in VHL patients treated with dovitinib.

### Statistical analysis

The study was designed to enroll 25 subjects. The trial design used a continuous Bayesian stopping rule based on toxicities and for lack of efficacy after 14 patients. Enrolling 14 patients would yield 83% power to detect the difference between the null hypothesis proportion of 5% response rate (PR + CR) and the alternative proportion, 30%, using an exact binomial test with a two-sided significance level of 10%. Because each individual VHL lesion is considered to have potential clinical significance in this patient population, independent analysis was carried out both on individual organ lesions and on a per-patient basis.
